# Aerobic Exercise, Training Dose, and Cardiorespiratory Fitness: Effects and Relationships with Resting Plasma Neurotrophic Factors in Alzheimer’s Dementia

**DOI:** 10.3390/jvd2030027

**Published:** 2023-09-01

**Authors:** Dereck L. Salisbury, Danni Li, Michael Todd, Ted K. S. Ng, Fang Yu

**Affiliations:** 1 School of Nursing, University of Minnesota, Minneapolis, MN 55455, USA; 2 School of Medicine, University of Minnesota, Minneapolis, MN 55455, USA; 3 Edison College of Nursing and Health Innovation, Arizona State University, Tempe, AZ 85281, USA; 4 Department of Internal Medicine & Rush Institute of Healthy Aging, Rush University Medical Center, Chicago, IL 60612, USA

**Keywords:** exercise, vascular factors, neurotrophic biomarkers, Alzheimer’s disease, cardiorespiratory fitness, dose-response

## Abstract

**Background:**

Vascular health is increasingly recognized for its roles in the pathogenesis and progression of Alzheimer’s disease (AD). The objective of this study was to investigate effects of exercise training, dose, and cardiorespiratory fitness (CRF) on neurotrophic factors in community-dwelling, older adults with mild-to-moderate AD dementia.

**Methods:**

This was a pilot blood ancillary study of the FIT-AD trial. Participants in the parent study were randomized to 6-month aerobic exercise (AEx) or stretching control. For this ancillary study, resting plasma brain-derived neurotrophic factor (BDNF), irisin, fibroblast growth factor-21 (FGF-21), and insulin-like growth factor-1 (IGF-1) biomarkers were assessed at baseline, 3, and 6 months. Estimates of within- and between-group effect sizes were calculated (Cohen’s d). Relationships of biomarker change with dose and CRF change were explored with multivariable linear regression and repeated measures correlations.

**Results:**

The sample (n = 26, 18 AEx/8 stretching) averaged 77.6 ± 6.9 years old, with the majority being male (65.4%), and non-Hispanic White (92.3%); between-group effect sizes were generally small except for irisin (d = −0.44)), AEx group relative to stretching group. Associations of dose and changes in CRF with changes in neurotrophic biomarker were weak (r^2^ ≤ 0.025).

**Conclusions:**

The effects of exercise on BDNF, irisin, IGF-1, and FGF-21 were heterogeneous in AD. Our findings need validation in future, adequately powered exercise studies in AD.

## 1. Introduction

Alzheimer’s disease (AD), currently afflicts 6.5 million Americans over the age of 65 years, and is projected to affect 13.8 million by 2060 [1]. AD is classically considered a neurodegenerative disease characterized by the presence of neurofibrillary tangles from hyperphosphorylated tau and accumulation of β-amyloid plaques in the brain [2], which culminates in a clinical milieu consisting of cognitive impairment, behavioral and psychological symptoms, and inability to perform activities of daily living [3].

However, as our understanding of AD pathophysiology has evolved, it is increasing realized that this devastating disease likely involves multifactorial pathologies, and in particular, support has accumulated for the view that vascular factors play a key patho-physiological role in the cognitive decline seen in AD [4,5]. This evidence is supported by post-mortem studies where vascular pathology has been concurrent in the majority of cases of clinically diagnosed AD [4]. Vascular alterations seen in AD are at the local (brain) and systemic levels. Local vascular pathological findings include increased intracranial atherosclerosis and cerebral amyloid angiopathy [6], while systemic changes collectively reduce cerebral blood flow [7]. The mechanisms driving the association between atherosclerosis (and other vascular alterations) and β-amyloid accumulation in AD are incompletely understood. One hypothesis is that this is the result of a repetitive and toxic accumulation of vascular inflammation and free radical-mediated oxidative stress, which promotes and exacerbates endothelial dysfunction that is destructive to the already deteriorating vasculature and brain [6]. On the molecular level, vascular health is modulated by many biomolecules including neurotrophic factors. Neurotrophic factors, classically named for their neurogenerative and neuroprotective properties [8], are increasingly studied for their pleiotropic, cardioprotective effects. Some of the more studied cardioprotective neurotrophic factors include brain-derived neurotrophic factor (BDNF), irisin (fibronectin type III binding domain-contain protein), insulin-like growth factor 1 (IGF-1), and fibroblast growth factor 21 (FGF-21) [9–12]. Collectively, these neurotrophic factors are thought to elicit their vascular health properties through the reduction of traditional cardiovascular disease (CVD) risk factors (i.e., hyperlipidemia, insulin resistance, and hypertension) as well as anti-inflammatory and antioxidant effects that mediate improvements in endothelial functioning and attenuate the atherosclerotic process [11,13,14]. The growing evidence of neuro- and cardioprotective effects of neurotrophic factors is further supported by findings that resting plasma neurotrophic factor levels are generally suppressed in persons with AD dementia [15,16] and atherosclerotic CVDs [17,18].

Coinciding with the growing evidence of vascular factors in AD pathogenesis, substantial data have also accumulated in support of the role exercise plays in the prevention and management of AD and age-related cognitive decline [19,20]. Exercise, particularly aerobic exercise (AEx), is known for its ability to improve vascular health physiologically and molecularly. Physiologically, exercise induces favorable changes in traditional CVD risk factors in parallel with direct effects related to improving endothelial function [21]. Molecularly, exercise provides a stimulatory effect for the secretion of neurotrophic factors from several tissue types (e.g., muscle, liver, adipose, endothelial cells), believed to be in response to increased metabolic demands [8]. Neurotrophic factors released in response to exercise include, but are not limited to, BDNF, irisin, FGF-21, and IGF-1 [8,22]. As exercise exerts beneficial effects on the vasculature through its stimulatory effect on several neurotrophic factors, these proteins are dually capable of inducing changes in the brain (neurogenesis and neuroplasticity) [22,23]. Therefore, exercise-induced neurotrophic factor release represents a plausible mechanism by which exercise may promote both cardiovascular and brain health, important for the management of AD dementia. Indeed, exercise, and in particular AEx, is now considered an important neuroprotective therapy based on subgroup findings from meta-analyses specific to cognitively intact older adults [24] as well as adults with mild cognitive impairment [25], mediated in part by increased resting plasma neurotrophic biomarkers.

However, the effect of AEx on resting plasma neurotrophic factor levels in AD dementia is understudied and is limited to two studies, which have focused only on BDNF [26,27] and IGF-1 [26]. Likewise, when looking at the effects of AEx as a therapy for chronic diseases, its pleiotropic effects are generally believed to be mediated in part by dose and cardiorespiratory fitness (CRF) [28], both yet to be studied in the context of neurotrophic factors in AD dementia. Hence, the primary objective of this pilot study was to derive preliminary estimates of within- and between-group effect sizes from a 6-month AEx or stretching intervention on resting plasma neurotrophic factors in older adults with mild-to-moderate AD dementia. Secondarily, it investigated the relationships of exercise dose and CRF change with resting plasma neurotrophic factors to help inform directions of future exercise RCTs. We expected that AEx would produce favorable trends in plasma neurotrophic biomarkers compared to stretching exercise in older adults with mild-moderate AD dementia. Additionally, exercise dose and CRF would be positively associated with change in resting plasma neurotrophic factor levels.

## 2. Materials and Methods

### 2.1. Design

This study used a cohort design and was the blood ancillary study of the FIT-AD trial [29]. The parent FIT-AD trial investigated the effects of 6-month AEx or stretching on cognition and hippocampal volume in community-dwelling older adults with mild-to-moderate AD [30]. The parent FIT-AD trial, which implemented a 2-parallel group design, randomized 96 participants to either an AEx or stretching control group using a 2:1 allocation ratio. The study statistician generated the randomization scheme, which was stratified by age (66–75, 76–85, and 85+), and used random permutated blocks of 3 and 6 participants. Investigators, with exception of the study statistician and data collectors, were blinded to group allocation [30]. The parent FIT-AD trial adhered to the Consolidated Standards of Reporting Trials (CONSORT) elements [31]. The findings from the parent FIT-AD trial (NCT01954550) pertaining to primary outcomes (effects of intervention on cognition) and secondary outcomes (effects of intervention on hippocampal volume) have been previously published [30].

**Figure 1.**
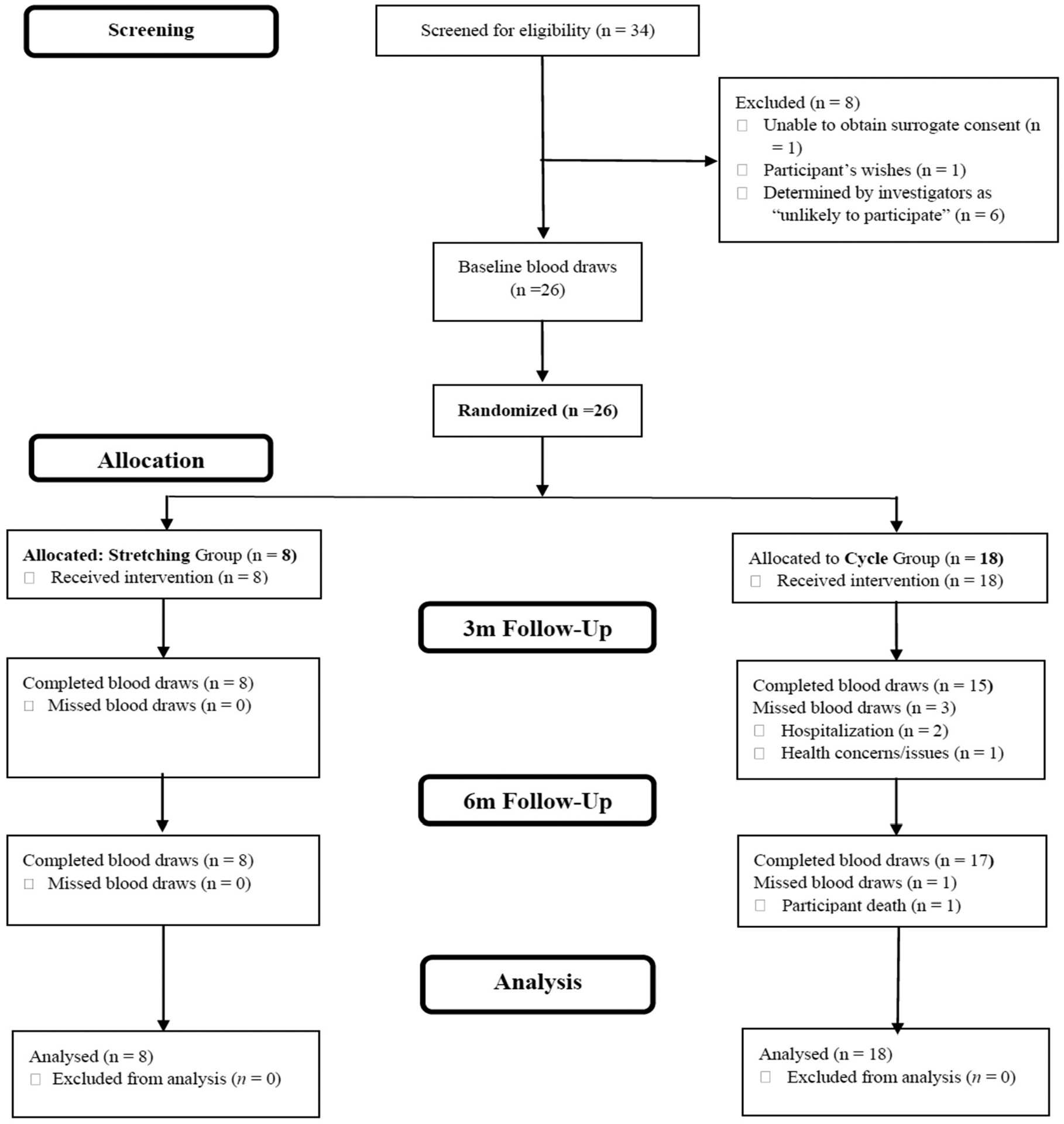
FIT-AD blood study CONSORT diagram.

This blood ancillary study was implemented during year 2 of the parent FIT-AD trial after 30 participants were already enrolled. Since the emphasis of this pilot study was to provide preliminary results to estimate effect sizes for power calculation for a future large-scale study, an a priori power analysis was not conducted. We anticipated 30 enrollees over the remaining years of the study and subsequently determined a target sample size of 25 after adjusting for a conservative attrition rate of 15% [29] (Figure 1). The targeted sample size was in accordance with sample size recommendations for pilot studies as previously published [32–34]. This ancillary blood study was approved by the university’s Institutional Review Board (IRB: #1508M77566). For participants who demonstrated the capacity to consent, written informed consent was obtained. Written assent and surrogate consent were obtained for participants who could not ethically provide informed consent. All procedures involving experiments on human subjects were done in accordance with the ethical standards of the Committee on Human Experimentation of the institution in which the experiments were done or in accord with the Helsinki Declaration of 1975.

### 2.2. Sample

To be eligible for the parent FIT-AD trial [30], participants were required to be community-dwelling, non-institutionalized older adults (over the age of 65) with a clinical diagnosis of AD dementia. Clinical diagnosis of AD dementia was verified by their primary care providers and by 3 clinician investigators (geriatric psychiatrist, neuropsychologist, and gerontological nurse practitioner) per 2011 diagnostic criteria. The degree of cognitive impairment required for study inclusion was mild-to-moderate (defined as a scores of 15–26 on the Mini-Mental State Examination [MMSE] and 0.5–2 on the Clinical Dementia Rating [CDR] scale). In addition, to be included in the parent FIT-AD trial, participants had to have physician clearance and be stable on AD drugs (i.e., taking for >1 month) if prescribed. The exclusion criteria included having a contraindication to exercise (as defined by the American College of Sports Medicine [ACSM]) [35] and having a non-AD cause of dementia/cognitive impairment (psychiatric condition [e.g., major depressive disorder], chemical dependency, or neurologic condition [e.g., Parkinson’s disease]) that was likely causative of the cognitive impairment.

Participants in the parent FIT-AD trial were required to meet additional eligibility criteria if they wished to participate in the ancillary blood study [29]. Interested participants had to (1) agree to adhere to the pre-sampling instructions for the 24 h preceding the scheduled blood collection; and (2) agree to donate 20 mL blood at each time point (60 mL total).

### 2.3. Experimental Protocol

All participants were encourage to complete three supervised sessions a week over 26 (72 sessions). All sessions were directly supervised in-person with a maximum interventionist-to-participant ratio of 1:3. Study interventionists monitored their exercise responses (heart rate [via heart rate monitor], rating of perceived exertion [RPE], ability to talk, and signs and symptoms). All sessions started at 30 min (including 5 min warm-up and 5 min cool-down) and were gradually lengthened to 60 min [30].

#### AEx

Participants completed an AEx program on recumbent cycle ergometers that implemented a linearly progressive, moderate-vigorous intensity protocol. Moderate-vigorous intensity was defined as 50–75% heart rate reserve (HRR) and was individually prescribed from results of the cardiopulmonary exercise test (CPET). A secondary measure of intensity utilized was RPE, where prescriptions progressively increased from 9 to 15 using the classic 6–20 Borg RPE scale [36]. The initial exercise prescription was set for 20 min at an intensity of 50–55% of HRR or RPE 9–11. By week 8 (24 sessions), participants had completed the ramp-up phase and then were in the maintenance phase and exercised for 50 min a session at a target intensity level of 70–75% HRR (or RPE 14–15) [30].

#### Stretching

Range of motion and flexibility training was prescribed as light (i.e., HRR ≤ 20%, RPE ≤ 8) with session frequency and duration matched to AEx group [30].

### 2.4. Outcome Variables

#### Blood Draws/Biomarkers

Blood sampling occurred at baseline, 3, and 6 month visits to the University of Minnesota Clinical and Translational Science Institute. All blood (plasma) samples were collected at least 48 h after the completion of last exercise session and between the hours of 0900–1200, in an attempt to account for collective peak plasma levels of the neurotrophic factors (i.e., diurnal variations) [37–41], while maintaining flexibility times for participant travel to get their blood drawn. Because this study was focused on the effects of chronic AEx on resting plasma neurotrophic biomarkers, blood draws were also conducted at least 48 h following completion of a last exercise session to ensure biomarker levels were not confounded by the effects of the last exercise session (i.e., acute effect of exercise) [42–45]. Samples were immediately placed on wet ice and transported to coauthor DL’s lab for processing. Briefly, to process and create aliquots of the samples, the laboratory technician inverted and centrifuged the plasma EDTA tubes for 15 min at 4 °C using a temperature-controlled centrifuge with a swing-out rotor at 1439 g. Immediately after completion, the tubes were removed from the centrifuge, were subsequently aliquoted, and stored in a freezer set to −80 °C [29].

Plasma-free and plasma total BDNF were measured using the Human Free BDNF and Human BDNF Quantikine ELISA kits (R&D Systems, Minneapolis, MN, USA), respectively. Plasma irisin was measured by Irisin ELISA kit (Phoenix Peptide, Burlingame, CA, USA). Plasma IGF-1 was measured on the Roche Cobas 8000 instrument. Plasma FGF-21 was measured using the Human Insulin-like Growth Factor Binding Protein 1 Quantikine ELISA kit and Human Fibroblast Growth Factor 21 Quantikine ELISA kit (R&D Systems, Minneapolis, MN, USA), respectively. Coefficient of variation (CV) for inter-assay CV (%) variations in plasma values were 1.7–4.8%, 2.1–4.3%, 9.5–15.7%, 1.3–2.1%, and 1.7–5.9% for BDNF-free, BDNF-total, irisin, IGF-1, and FGF-21, respectively.

#### Exercise Dose

To calculate training dose, we implemented a practical heart rate monitor-based measure (heart rate physical activity score HRPAS) [46]. A session HRPAS was quantified following each session, where the session HRPAS reflected the product of intensity (average session HRR achieved) and session duration (min). The individual HRPAS from each session was then summed to give a cumulative HRPAS (i.e., exercise dose).

#### CRF

Cardiopulmonary exercise test. Participants completed a CPET on a recumbent cycle ergometer (Precor 842i, Woodenville, WA, USA) at the University of Minnesota’s Laboratory of Clinical Physiology, as previously published [47]. Expired gases were measured continuously by a respiratory mass spectrometer (MGA 1100, Beck’s Physiological Systems, St. Louis, MO, USA), with breath-by-breath analysis averaged over 30 s intervals. Briefly, participants cycled at 50–60 RPM and every 3 min, the resistance of the cycle was increased to promote volitional fatigue. Participants were instructed to cycle until they were no longer able to maintain cycling rate (i.e., volitional fatigue). Predetermined termination criteria in the absence of volitional fatigue included (1) achieving the ACSM criteria for achieving a maximal effort [35]; or (2) ACSM relative or absolute indications to terminate based on clinical signs and symptoms [35]. VO_2peak_ was defined as the median oxygen consumption during the last 30 s before cessation of exercise [47].

### 2.5. Statistical Analyses

Descriptive statistics with means/standard deviations (SD) and frequencies/percentage were used to summarize the characteristics (clinical and demographic) of the participants at baseline. Statistical assumptions were checked prior to analysis. The chi-square test and independent samples *t*-test were used for categorical measures and continuous measures, respectively, to test differences in baseline characteristics between the stretching and AEx groups.

Effect size estimates for between-group differences in change in each biomarker from baseline to follow-up assessment (either 3-month or 6-month) were computed by first obtaining a partial r^2^ corresponding to the group (AEx vs. stretching) effect using a pair of nested regression models (Model 1: follow-up biomarker value predicted from baseline biomarker value; Model 2: follow-up biomarker value predicted from baseline biomarker value and study group). The difference in model R^2^s (Model 2 R^2^—Model 1 R^2^) was then converted to a standardized mean difference (Cohen’s d). Estimates of within-group effect sizes (standardized between-time point differences) for change in biomarkers from baseline to follow-up were derived from the paired sample *t*-test comparing follow-up values to baseline values within each study group.

To examine the associations between measures of exercise dose received and change in biomarker levels, we estimated a set of multivariable linear regression models, with total dose incurred over the course of the intervention (3 and 6 months) predicting each biomarker level at 3 and 6 months, adjusting for the corresponding biomarker level at baseline. To examine associations between change in CRF and changes in biomarkers, we used repeated measures correlations. Three correlations were computed for each CRF-biomarker combination: correlation between changes in CRF and the biomarker from baseline to the 3-month assessment; between changes in CRF and the biomarker from baseline to 6 months; and between changes in CRF and the biomarker across all three time points. The corresponding r^2^ values (obtained by squaring the repeated measures correlation coefficients) were also computed. Analyses were conducted in R 4.1.3 using the lm function for multivariable regression models, the r_to_d and t_to_d functions of the effectsize package for effect size estimation, and the rmcorr package for repeated measures correlations.

## 3. Results

### 3.1. Sample and Intervention Descriptors

In total, 44 consecutive potential participants of the parent FIT-AD trial received recruitment materials for this ancillary blood study via email. Of the 44 that received the recruitment materials, 10 did not respond. For those who responded (n = 34), we screened out 8. Of the 8 who did not pass the screening process, 6 were understood to be unlikely to participate (defined as unlikely for successful blood collections due to (1) time burden, (2) caregiver burden, or (3) travel barriers). Of the 26 enrolled participants, 8 were in the stretching group and 18 were in the AEx group (Figure 1). The sample was 35% female and 92% non-Hispanic White with a mean age of 77.6 (6.9) years and MMSE score of 21.6 (3.3). There were no significant differences between the two groups at baseline regarding demographic and clinical descriptors (Table 1). Adherence (exercise session attendance) was not significantly different between groups (collective session attendance 84.8%).

### 3.2. Plasma Neurotrophic Biomarker Changes

Table 2 provide summaries (mean and SD) of resting plasma neurotrophic biomarkers by group at baseline, 3 months, and 6 months, as well as between-group effect sizes. Between-group effect sizes were negligible with the exception of moderate effects for baseline to 3- and 6-month differences in irisin; both time points trended towards a decrease in biomarker for the AEx group relative to the stretching group. Table 3 summarizes within-group differences in resting neurotrophic biomarkers using baseline as reference (3 or 6 months: baseline), effect sizes, and *p*-values. The within-group effect sizes were mostly negligible (ds < 0.20) except for moderate effect sizes for baseline to 6-month differences in BDNF-free (d = −0.48) for the AEx group.

### 3.3. Exercise Dose and Biomarker Change

Associations between 3-month exercise dose and 3-month change in biomarkers were negative and quite weak (r^2^ ≤ 0.025), with several r^2^ values near zero with the exception of BDNF-free (r^2^ = 0.04). However, the trajectory of the dose-response relationship improved at 6 months for each of the biomarkers. Despite this change in trajectory, the associations between 6-month exercise dose and 6-month biomarker change remained quite weak (r^2^ ≤ 0.025) (Table 4).

### 3.4. Change in CRF and Biomarker Change

Associations of change in CRF with 3- and 6-month biomarker change were generally weak (r^2^s = 0.000–0.081; median r^2^ = 0.025) (Table 5). The two strongest associations were the negative association between baseline to 3-month changes in CRF and irisin (r = −0.284), indicating that as fitness increased, irisin levels decreased, and the positive association between baseline to 6-month changes in CRF and FGF-21 (r = 0.272), indicating that as fitness increased, so did FGF-21 levels.

## 4. Discussion

Blood biomarkers are increasingly recognized as important measurements to evaluate the therapeutic effects of interventional therapies (including exercise) for AD [48]. This pilot study was unique in exercise-AD research as it (1) employed a stretching control group in a study with resting plasma neurotrophic factor outcomes, (2) investigated a dose-response relationship using a fluid measure of exercise dose, and (3) used a gold standard laboratory measurement of CRF performed on a cycle ergometer (not commonly utilized in AD research). One preliminary finding was that the between-group effect sizes for testing plasma BDNF and IGF-1 were negligible (<0.20). These between-group effect sizes are smaller in magnitude relative to the findings from the limited number of AEx studies involving persons with AD dementia [26,27]. First, Stein and colleagues showed small-to-moderate effects of 12 weeks’ AEx compared to usual-care control on resting plasma BDNF (SMD = 0.31, 95%CI [−0.45, 1.05]) and IGF-1 levels (SMD = −0.61, 95%CI [−1.29, 0.10]) in 34 participants with mild-to-moderate AD dementia [26]. Likewise, a second study by Enette et al. with 51 participants with mild-to-moderate AD randomized to 9 weeks of moderate intensity AEx, high-intensity interval training, or educational control showed a negative effect on plasma BDNF (SMD = −1.4, 95%CI [−2.02, −0.78]) [27].

To our knowledge, our study is the first AEx-focused RCT investigating the effects of exercise on resting plasma irisin in persons with mild-to-moderate AD dementia. Three meta-analyses have looked at the effects of AEx on resting plasma irisin in cognitively intact adults. Two found that irisin levels decrease following AEx compared to controls (d = −0.64; 95%CI [−1.32, 0.04]) [49], (Hedge’s g = −0.18; 95%CI [−0.73, 0.37]) [50]. The third showed that irisin did not change following AEx (0.01 μg/mL [95%CI, −0.03, 0.02], *p* = 0.60) [51]. The moderate effect size for between-group difference (and its direction) found in our study is in agreement with these aforementioned findings [49]. Currently, the reasoning and importance of these effects can only be speculated. Irisin is a well-known myokine that peaks within a few hours following acute exercise, has a short half-life, and returns to baseline within 24 h [8]. However, irisin is also released from other tissues including adipose (i.e., adipokine) [8] and resting plasma levels have been shown to decrease after body fat reduction [52]. It is speculated that AEx-mediated reduction in resting plasma irisin may be induced by reduced adiposity [50]. In this study, weight and body mass index (BMI) change were non-significant in both groups and the correlation between changes in resting irisin and changes in BMI were also non-significant at both 3 (r = −0.09; *p* = 0.68) and 6 (r = 0.04; *p* = 0.84) months. Though BMI has known limitations as a surrogate measure of adiposity, we were unable to directly assess fat mass with gold standard techniques. Additionally, other research suggests that irisin levels positively correlate with insulin resistance [53]. This relationship is important given the well-noted positive effects of AEx on insulin resistance and blood glucose homeostasis [54], both of which are important contributors to vascular and brain health [55]. Future research efforts should investigate the effects of AEx on resting plasma irisin and its relationships with adiposity, insulin resistance, and blood glucose homeostasis in persons with AD dementia.

The findings from this study add to the evidence that exercise training may have highly variable effects on resting neurotrophic biomarker levels. The reason for this heterogeneity in response may be due to several factors including variability in exercise prescription used in studies, and clinical (i.e., healthy adults, obesity, neuropsychiatric conditions, and neurodegenerative conditions) and racial/ethnic characteristics of study participants. In addition, some researchers speculate that the measurement of change in both resting plasma neurotrophic factor levels and the acute response to exercise stimuli following an exercise training intervention should be measured to evaluate the neurotrophic effect of exercise, given that plasma neurotrophic factors (including the ones measured in this study) peak and return to baseline within 24 h of completing an acute bout (session) of exercise [43,56–58]. Regardless, it is evident that more research is needed to establish if resting plasma neurotrophic levels can be positively modulated in persons with AD dementia following participation in exercise training.

We also evaluated the dose-response relationships between AEx intervention and resting neurotrophic factor levels yet to be studied in persons with AD dementia. Exercise dose is a product of exercise frequency, intensity, session length (duration), and program length); however, these components are classically looked at individually rather than collectively regarding their potential influence on training response. Subgroup analyses from published meta-analyses that included only adults without neurodegenerative conditions have suggested that components of exercise-dosing metrics on resting plasma BDNF levels including frequency [59], intensity [59], session duration [59], and program length [59,60] are not associated with degree of change in resting plasma BDNF levels in cognitively intact adults. Similar investigations of exercise dose on resting plasma neurotrophic factors in AD dementia or in general on non-BDNF neurotrophic levels have yet to be conducted. Likewise, few studies have investigated the relationship between CRF changes, measured by VO2Peak, with resting plasma neurotrophic biomarker levels changes following AEx intervention. Our findings align with those recently published by Allard and colleagues, who showed no significant correlation between changes in resting plasma BDNF and changes in VO2Peak (r = 0.292; *p* = 0.20) in participants with mild cognitive impairment [61]; however, investigations in persons with AD dementia are lacking. To our knowledge, there has yet to be a study that has assessed the relationship between changes in VO_2Peak_ with changes in resting FGF-21, irisin, and IGF-1.

The FIT-AD ancillary blood study has several weaknesses that warrant discussions in addition to the small sample size inherent to the nature of a pilot study. One potential weakness is the use of a stretching control instead of a sedentary or usual-care control group. However, the choice to utilize a stretching control group reflected the primary aims (cognitive function) of the parent FIT-AD trial to control for the Hawthorne effect and social interaction between participants and between participants and exercise interventionists [30]. Future trials could include a usual-care control on top of a stretching control to parse out the interventions’ differential effects. Likewise, a lack of a non-dementia control group may be perceived as a study weakness. The decision to refrain from the use of a non-dementia control group in the parent FIT-AD trial was based on two main factors: (1) older adults with AD have more chronic conditions and multimorbidity [62]; and (2) they exhibit prevalent behavioral and psychological symptoms of dementia, both of which can affect their exercise participation and exercise interventions [63]. Hence, comparing older adults with AD dementia to non-AD dementia peers is to compare two different populations who may react to exercise differently, which can also be perceived as a weaker trial design. The generalizability of this study may be impacted by the high percentage of participants (96.2%) who had at least one APOE e4 allele, which is higher than the generally reported 40–65% seen in all AD cases [64]. In animal models, APOE e4 has been shown to promote impaired BDNF processing compared to APOE e2 and APOE e3 [65]. However, to our knowledge, the effects of AEx on irisin, FGF-21, and IGF-1 in human APOE e4 carriers vs. non-carriers has yet to be studied. Hence, the clinical significance of this high level of APOE e4 carriers in this sample pertaining to a “neurotrophic response to exercise” is unknown and represents another future direction of study. The predominantly White Caucasian sample also limits the generalizability of this pilot study. The lack of a diverse sample has been reported as a shortcoming in other neuroscience and AD-based research [66], and must be addressed in future studies [67]. Lastly, the ancillary blood study was not powered to test the statistical significance of intervention effects or other associations; accordingly, the focal results were estimates of effect sizes (standardized mean differences and r^2^ values). Thus, reported *p*-values should be interpreted with appropriate caution.

Despite the pilot and preliminary nature of this study, the study design and findings will inform the refinement of the design and conceptual framework for future fully powered studies investigating the peripheral mechanisms through which exercise training modulates brain function and cognition. The foremost strength of this ancillary blood study was that the parent FIT-AD trial [30] was a meticulously controlled study designed to evaluate the effects AEx on cognition and hippocampal volume in persons with AD dementia. Secondly, this study used a novel and real-life approach to assess exercise dose, as discussed previously [46]. Likewise, we employed a gold standard measurement of CRF (VO_2peak_ from CPET) for investigating the effects of change in CRF on resting plasma neurotrophic biomarkers. This is important given the negative influence of motor dysfunction on gait in persons with AD dementia [68] and therefore potential negative influence on walking-based CRF field tests.

## 5. Conclusions

Vascular factors are increasingly recognized for their roles in the pathogenesis and progression of AD, with neurotrophic factors being an important link between vascular health, exercise, and cognition. Findings from this study suggested that the effects of exercise on BDNF, irisin, IGF-1, and FGF-21 may be heterogeneous in older adults with mild-to-moderate AD dementia. The weak linear associations of exercise dose and CRF changes with changes in resting neurotrophic biomarkers may be attributable to this kind of heterogeneity. This study provides preliminary data that can inform selection of candidate neurotrophic biomarkers, study design considerations, and samples size estimates for fully powered exercise studies investigating peripheral plasma neurotrophic factors as potential mechanisms for exercise’s effects in AD dementia.

## Figures and Tables

**Table 1.** Demographic and clinical variables.

	All (n = 26)	Cycling (n = 18)	Stretch (n = 8)	T Value or χ^2^	*p*
Age	77.6 (6.9)	76.8 (7.6)	79.3 (5.5)	−0.81	0.423
Sex (Female)	9 (34.6)	7 (38.9)	2 (25.0)	0.472	0.492
Race/Ethnicity				2.72	0.256
non-Hispanic White	24 (92.3)	17 (94.4)	7 (87.5)		
Hispanic White	1 (3.8)	1 (5.5)	-		
Black American	1 (3.8)	-	1 (12.5)		
Education (years)	15.4 (2.9)	15.9 (3.2)	14.5 (2.5)	1.05	0.361
APOE genotype				2.88	0.224
E2/E3	3.8%	5.6%	0%		
E3/E4	42.3%	33.3%	62.5%		
E2/E4	26.9%	33.3%	12.5%		
E4/E4	26.9%	27.8%	25.0%		
BMI	27.7 (4.4)	26.8 (4.1)	29.7 (4.7)	−1.58	0.126
MMSE	21.6 (3.3)	21.3 (3.7)	22.3 (2.3)	−0.96	0.348
CVD	6 (23.1)	4 (22.2)	2 (25.0)	FET	0.651
Beta blocker	1 (3.8)	-	1 (12.5)	FET	0.308
AD medications	17 (65.4)	11 (61.1)	6 (75.0)	FET	0.413
VO_2Peak_ (mL/kg/min)	18.3 (4.6)	18.4 (5.0)	18.0 (3.8)	0.20	0.842

APOE; apolipoprotein E: BMI; body mass index: MMSE; mini mental state examination: CVD; cardiovascular disease: AD; Alzheimer’s dementia: VO_2Peak_; peak oxygen consumption: FET: Fischer exact test.

**Table 2.** Between-group differences (cycling minus stretching) in biomarker change from baseline to 3- and 6-month follow-up.

					3 Months vs. Baseline			6 Months vs. Baseline		
Biomarker	Group	Baseline	3 Months M (SD)	6 Months M (SD)	Mean Difference^a^ (95% CI)	d^b^	*p*	Mean Difference^a^ (95% CI)	d^b^	*p*
BDNF-free (pg/mL)	Cycling	999.50 (722.30)	1481.00 (1273.37)	1201.44 (958.92)	−230.13 (−1443.59, 983.34)	−0.18	0.696	18.82 (−854.88, 892.52)	0.02	0.965
	Stretching	1408.38 (845.63)	1824.86 (1095.39)	1671.75 (1603.10)						
BDNF-total (pg/mL)	Cycling	1166.67 (840.37)	1531.87 (1520.52)	1240.75 (730.17)	97.666 (−1197.24,1392.57)	0.07	0.876	−142.71 (−1051.38,765.96)	−0.14	0.747
	Stretching	1560.25 (957.02)	1629.43 (863.76)	1709.25 (1672.96)						
Irisin (ng/mL)	Cycling	5.00 (2.69)	4.39 (0.52)	4.35 (0.53)	−0.17 (−0.57, 0.23)	−0.40	0.395	−0.186 (−0.56, 0.19)	−0.44	0.320
	Stretching	4.27 (0.49)	4.52 (0.46)	4.44 (0.49)						
FGF-21 (pg/mL)	Cycling	299.50 (213.60)	261.53 (164.04)	258.12 (128.44)	−60.52 (−218.49, 97.46)	−0.37	0.582	14.56 (−109.498,138.612)	0.11	0.990
	Stretching	343.88 (266.26)	328.14 (278.15)	264.75 (153.87)						
IGF-1 (pg/mL)	Cycling	98.18 (31.68)	95.91 (29.34)	100.96 (36.98)	−4.05 (−19.17,11.06)	−0.25	0.190	−0.08 (−12.74,12.59)	−0.01	0.993
	Stretching	100.35 (38.83)	103.11 (37.51)	102.05 (39.51)						

a Model-estimated between-group mean difference (cycling minus stretching) in change from baseline.

b Standardized between-group mean difference (cycling minus stretching) in change from baseline. BDNF: brain-derived growth factor; FGF-21: fibroblast growth factor 21; IGF-1: insulin-like growth factor 1.

**Table 3.** Within-group effect size estimates for change in biomarkers from baseline to 3- and 6-month follow-up.

		Cycling Group 3 Months vs. Baseline				Cycling Group 6 Months vs. Baseline		
Biomarker	n	Mean Difference (95% CI)	d	*p*	n	Mean Difference (95% CI)	d	*p*
BDNF-free (pg/mL)	15	−385.40 (−1155.49, 384.69)	−0.29	0.301	16	−250.00 (−670.22,170.22)	−0.33	0.224
BDNF-total (pg/mL)	15	−285.93 (−1170.96, 599.09)	−0.19	0.500	16	−148.69 (−556.59, 259.21)	−0.20	0.449
Irisin (ng/mL)	15	−0.01 (−0.25,0.22)	−0.03	0.899	16	0.05 (−0.17,0.27)	0.12	0.635
FGF-21 (pg/mL)	15	3.27 (−87.88, 94.41)	0.02	0.940	16	−5.75 (−90.30, 78.80)	−0.04	0.887
IGF-1 (pg/mL)	15	0.49 (−9.23,10.22)	0.03	0.915	17	−1.60 (−8.11, 4.90)	−0.13	0.609
		Stretching Group 3 Months vs. Baseline				Stretching Group 6 Months vs. Baseline		
Biomarker	n	Mean difference (95% CI)	d	*p*	n	Mean difference (95% CI)	d	*p*
BDNF-free (pg/mL)	7	−339.00 (−1413.98, 735.98)	−0.32	0.470	8	−263.38 (−1201.54,674.79)	−0.25	0.528
BDNF-total (pg/mL)	7	34.14 (−526.14, 594.43)	0.06	0.886	8	−149.00 (−1270.22, 972.22)	−0.12	0.763
Irisin (ng/mL)	7	−0.20 (−0.63,0.23)	−0.47	0.294	8	−0.17 (−0.52, 0.18)	−0.42	0.299
FGF-21 (pg/mL)	7	−56.43 (−199.19, 86.33)	−0.39	0.371	8	79.12 (−125.14, 283.39)	0.35	0.390
IGF-1 (pg/mL)	8	−2.76 (−17.59,12.06)	−0.17	0.673	8	−1.70 (−15.52, 12.12)	−0.11	0.780

BDNF: brain-derived growth factor; FGF-21: fibroblast growth factor 21; IGF-1: insulin-like growth factor 1.

**Table 4.** Associations between dose and biomarker values at 3- and 6-month follow-up, adjusting for baseline biomarker values.

	3-Month Dose (Intensity-Minutes)			6-Month Dose (Intensity-Minutes)		
Biomarker	b	r^2^	*p*	b	r^2^	*p*
BDNF-free (pg/mL)	−0.726 (−2.419, 0.968)	0.04	0.381	−0.052 (−0.645, 0.540)	0.00	0.856
BDNF-total (pg/mL)	−0.440 (−2.240,1.359)	0.01	0.614	−0.159 (−0.765, 0.448)	0.01	0.592
Irisin (ng/mL)	0.000 (−0.001, 0.001)	0.00	0.840	0.000 (0.000,0.000)	0.02	0.461
FGF-21 (pg/mL)	−0.066 (−0.280, 0.149)	0.01	0.531	0.000 (−0.083, 0.083)	0.00	0.999
IGF-1 (pg/mL)	−0.008 (−0.029, 0.012)	0.01	0.396	0.003 (−0.006,0.011)	0.00	0.557

Note. b = unstandardized regression coefficient. r^2^ = proportion of variance in biomarker measure at follow-up accounted for by dose. BDNF; brain-derived growth factor: FGF-21; fibroblast growth factor 21: IGF-1; insulin-like growth factor 1.

**Table 5.** Longitudinal correlations between cardiorespiratory fitness and biomarker values for baseline to 3 months, baseline to 6 months, and across all time points.

	Baseline-3 Months		Baseline-6 Months		All Time Points	
Biomarker	r	*p*	r	*p*	r	*p*
BDNF-free (pg/mL)	−0.016 (−0.627, 0.554)	0.942	−0.032 (−0.484, 0.342)	0.881	−0.133 (−0.451,0.197)	0.372
BDNF-total (pg/mL)	−0.134 (−0.631, 0.371)	0.542	−0.100 (−0.540, 0.297)	0.634	−0.223 (−0.427, −0.006)	0.133
Irisin (ng/mL)	−0.284 (−0.718, 0.308)	0.189	0.019 (−0.349, 0.535)	0.927	−0.125 (−0.406, 0.250)	0.403
FGF-21 (pg/mL)	0.038 (−0.604, 0.634)	0.860	0.272 (−0.018, 0.572)	0.179	0.135 (−0.161, 0.420)	0.356
IGF-1 (pg/mL)	0.241 (−0.262, 0.523)	0.268	0.013 (−0.612, 0.425)	0.951	0.073 (−0.311, 0.280)	0.624

Note. r = repeated measures correlation between biomarker measure and VO2peak. VO2peak; peak oxygen consumption: BDNF; brain-derived growth factor: FGF-21; fibroblast growth factor 21: IGF-1; insulin-like growth factor 1.

## Data Availability

All data generated or analyzed during this study are included in this publication.
